# Vernacular dominance in folk taxonomy: a case study of ethnospecies in medicinal plant trade in Tanzania

**DOI:** 10.1186/1746-4269-11-10

**Published:** 2015-02-19

**Authors:** Joseph Otieno, Siri Abihudi, Sarina Veldman, Michael Nahashon, Tinde van Andel, Hugo J de Boer

**Affiliations:** Institute of Traditional Medicine, Muhimbili University of Health and Allied Sciences, P.O. Box 65001, Dar es Salaam, Tanzania; Department of Organismal Biology, Evolutionary Biology Centre, Uppsala University, Norbyvägen 18D, SE-75236 Uppsala, Sweden; Naturalis Biodiversity Center, Darwinweg 4, Postbus 9517, 2300 RA Leiden, The Netherlands; The Natural History Museum, University of Oslo, P.O. Box 1172, NO-0318 Oslo, Norway

**Keywords:** Vernacular names, Medicinal plants, Wildlife trade, Tanzania, Sub-Saharan Africa, Traditional medicine, Folk taxonomy

## Abstract

**Background:**

Medicinal plants are traded as products with vernacular names, but these folk taxonomies do not always correspond one-to-one with scientific plant names. These local species entities can be defined as ethnospecies and can match, under-differentiate or over-differentiate as compared to scientific species. Identification of plant species in trade is further complicated by the processed state of the product, substitution and adulteration. In countries like Tanzania, an additional dimension to mapping folk taxonomies on scientific names is added by the multitude of ethnicities and languages of the plant collectors, traders and consumers. This study aims to elucidate the relations between the most common vernacular names and the ethnicity of the individual traders among the medicinal plant markets in Dar es Salaam and Tanga regions in Tanzania, with the aim of understanding the dynamics of vernacular names in plant trade.

**Methods:**

A total of 90 respondents were interviewed in local markets using semi-structured interviews. The ethnicity of each respondent was recorded, as well as the language of each ethnospecies mentioned during the interviews. Voucher collections and reference literature were used to match ethnospecies across languages.

**Results:**

At each market, the language of the majority of the vendors dominates the names for medicinal products. The dominant vendors often represent the major ethnic groups of that region. Independent of their ethnicity, vendors offer their products in the dominant language of the specific region without apparently leading to any confusion or species mismatching.

**Conclusions:**

Middlemen, traders and vendors adapt their folk classifications to those of the ethnic groups of the region where they conduct their trade, and to the ethnicity of their main customers. The names in the language of the traders are not forgotten, but relegated in favor of the more salient names of the dominant tribe.

**Electronic supplementary material:**

The online version of this article (doi:10.1186/1746-4269-11-10) contains supplementary material, which is available to authorized users.

## Background

In Africa, traditional healers and remedies made from plants play an important role in healthcare for millions of people [1, 2]. The often-quoted World Health Organization estimate that up to 80% of population depends on traditional medicine for their primary healthcare [3], is particularly apt for Tanzania where traditional medicine is a thriving trade in both rural and urban areas [4–8]. An estimated 5400 plant species are used in traditional medicine in Africa [9]. Estimates of the number of species used in Tanzania vary from 405 [10] to 1100 [11], although many authors agree that too little is currently known [6, 12].

Studying medicinal plants traded in markets has been used to quantify the commercialization and utilization of natural products, as these mirror a region’s culture, and can give a rapid insight into traditions, salient health problems and the importance of herbal medicine in local health care [13–21]. In addition, surveys of traded medicinal products can reveal the diversity and prevalence of these items [14, 15] and can help to estimate the total traded species richness [16, 17, 20, 21]. Nevertheless, some parts of the local pharmacopeia may be overlooked, and it has been asserted that species entering commercial trade represent a 'short-list’ of a far greater diversity of species used for subsistence in rural areas [13].

The rich floral biodiversity of Tanzania with over 11,000 species [22], the estimated number of 5400 species used in traditional medicine in Africa [9], and species diversity in markets in South Africa [14], Benin [20], Gabon [23], Ghana [21] and Morocco [18] suggest that between 200–800 species could be traded in Tanzania. However, published structured quantitative research on medicinal plant trade in the markets of Tanzania is so far lacking, and less than 200 plant species have been documented from trade [12, 24–27]. The two main cities trading wild-crafted medicinal plant products in Tanzania are Dar es Salaam with approximately 70 vendors, and Tanga with ca. 50 vendors [24, 25, 28], but the actual numbers are probably significantly higher. Commercial trade is said to be one of the largest threats to wild medicinal plant populations in Tanzania [24], and vendors in both markets have reported the coastal districts, as well as the Uluguru and Usambara mountains, as the main sources of plant material [24]. The main sources of wild-crafted medicinals coincide with the locations of some of the most threatened biodiversity hotspots in Africa: the Coastal Forest of Eastern Africa and Eastern Afro-montane hotspots [29].

If Tanzania is to provide a sustainable supply of herbal medicine in the future, it needs to know which species are in danger of being overexploited. Knowing the medicinal plant species in trade is a prerequisite for developing a system of controlled sustainable exploitation. Priority species for conservation would be those that are wild-harvested in large quantities from vulnerable habitats and reported to be increasingly difficult to obtain. Identification of medicinal plants in trade is complicated, as most material is traded in dried, shredded or powdered form [7, 25, 30].

Medicinal plants are traded as products with local names in folk taxonomies [31]. These local species entities can be defined as ethnospecies and can match, under-differentiate or over-differentiate as compared to scientific species. Under-differentiation in ethnospecies is the case when names are being applied to entire genera or unrelated species [13, 31], while over-differentiating happens beyond the scientific species level to distinguish intraspecific morphological or pharmacological characters, such as shape, colour and taste [32]. Even hallucinogenic experiences resulting from taking certain medicinal plants are used as properties for over-differentiation of plant species [33]. Other processes that complicate matching ethnospecies and scientific names of products in trade are substitution and adulteration. Adulteration is the intentional replacement of the intended species for another, while substitution is more broadly defined and can include slow general substitution of one species for another due to overharvesting and depletion of the original species [18, 30]. Substitution and adulteration are not only challenging for scholars of folk taxonomies, but can also be confusing for traders [19] and customers [30]. In Tanzania, as elsewhere in Africa, an additional dimension to mapping folk taxonomies on scientific names is added by the multitude of ethnicities and languages of the plant collectors, traders and consumers [34, 35].

This study aims to elucidate the origins of the vernacular names used for the most common ethnospecies in the Tanzanian medicinal plant trade. Our null hypothesis was that traders use the vernacular names in their own language for the ethnospecies they trade, while our alternative hypothesis was that traders adjust the names to those of the dominant group in the area in which they trade. The following research questions are posed: 1) What are the ethnicities of medicinal plant traders in Dar es Salaam and Tanga?; 2) What names do the traders use for the products in their stalls?; 3) What are the most likely scientific names for each of the ethnospecies?; 4) Which vernacular name(s) are dominant per ethnospecies?; and 5) What language dominates the medicinal plant trade and why?

## Methods

### Study area

The study was carried out in Dar es Salaam and Tanga regions from August 2013 to mid-April 2014. Dar es Salaam city and Tanga town are the main hubs of the medicinal plant trade in Tanzania [7, 25, 28]. Plant materials are supplied to here from the surrounding regions of Arusha, Iringa, Lindi, Manyara, Morogoro, and Pwani. These regions include two biodiversity hotspots that are recognized internationally for their high species diversity and endemicity—the Coastal Forests of East Africa and the Eastern Afromontane Forests [29], which are important sources of commercially-traded medicinal plants [7]. These coastal trade cities are melting pots of ethnicity, culture and religion [7, 36].

### Market survey

The survey focused on the main areas of medicinal plant trade of Dar es Salaam city and Tanga town, these included Kariakoo market in Dar es Salaam and various streets in Tanga town. In these areas traders are specialized in medicinal plant trade and these businesses rely solely on its commerce. Additional vendors, traditional healers and herbal specialists were surveyed in their shops in other markets, sitting along high traffic thorough ways, and walking around the bus stations, traffic intersections and ferry terminals peddling their medicinals. Most vendors stock their supplies crudely on shelves, tables, the ground, cloths or plastic tarps, while others offer processed mixtures in bottles, vials and plastic containers. A total of 90 respondents were interviewed in local markets using semi-structured interviews. The ethnicity of each respondent was recorded, as well as the language of each ethnospecies mentioned during the interviews.

The interview questionnaires were submitted to the Muhimbili University of Health and Allied Sciences (MUHAS), but no ethical review committee permission was needed. All participating vendors of medicinal plants were informed of the objectives of our research project prior to commencement of the interviews, and those that agreed to participate were interviewed.

### Botanical collections and identification

Voucher collections were collected and stored at the herbarium of the Institute for Traditional Medicine, Muhimbili University of Health and Allied Sciences. Botanists from the Muhimbili University of Health and Allied Sciences, Dar es Salaam University and National Herbarium of Tanzania aided in identifying the voucher samples. Specific collection trips were organized with willing traders and collectors to find botanical vouchers if identifications of the vouchers were ambiguous. Reference literature was used to match ethnospecies across languages [28].

### Data analysis

Spreadsheets of respondent data from each interview were compiled into a master table to identify and sort matching species and enumerate the frequency that each species was mentioned. After matching vernacular names and orthographic variation, a total of 207 ethnospecies were identified, of which 124 could be matched with scientific names. The remaining 83 ethnospecies were unidentifiable using either morphology-based identification or vernacular name-guided morphology-based identification. A total of 305 medicinal products were collected as vouchers, including 222 single-ingredient products and 83 multispecies mixtures. Ethnospecies that were reported by 10 or more respondents (19 in total) were analyzed to study vernacular dominance. All ethnospecies were scored by the language in which they were reported during the interview, and could either match the ethnicity of the informant (score 1 in Additional file 1) or differ (score 0 in Additional file 1).

## Results

A total of 90 vendors were interviewed: 79 in Dar es Salaam and 11 in Tanga. Respondents belonged to 18 ethnicities or groups and originated from 17 out of Tanzania’s 22 mainland regions (Table 1). A total of 124 species were identified from 207 reported ethnospecies. The data included 305 vouchered products, including 222 single ingredients and 83 mixtures. These vouchers could include a number of redundancies due to vernacular names in different languages. DNA barcoding of these unknowns could enable identification [18, 19, 37], but this remains to be done.

**Table 1:** **Ethnicity and origin of herbal vendor informants**

Reported	Ethnologue ^1^	Region	Number of informants
Bushaidi	*	Zanzibar	1
Digo	Digo	Tanga	1
Hehe	Hehe	Iringa; Morogora; Dodoma; Mbeya	1
Kwere	Kwere	Pwani; Morogoro	37
Maasai	Maasai	Arusha; Tanga; Kilimanjaro; Manyara	17
Mwera	Mwera	Lindi	1
Ndengereko	Ndengereko	Pwani; Morogoro	1
Ngunya	*	Zanzibar	2
Nyaturu	Nyaturu	Singida	1
Nyiramba	Nilamba	Singida	1
Pangwa	Pangwa	Iringa	1
Rangi	Langi	Dodoma	2
Sambaa	Shambala	Tanga	11
Segeju	Segeju	Tanga	1
Shirazi	*	Zanzibar	1
Sukuma	Sukuma	Shinyanga; Mwanza; Kagera; Tabora; Singida; Kigoma; Mara	1
Zaramo	Zaramo	Pwani	8
Zigua	Zigula	Tanga; Pwani; Manyara	2
		**Total number of informants**	**90**

^1^Ethnologue. Languages of the World. http://www.ethnologue.com/ *Groups from Zanzibar not present in Ethnologue.

Results for the 19 most salient ethnospecies were summarized in pie charts per species, including one figure showing the number of matching and non-matching names, one figure showing the language of the matching names and one figure showing the language of the non-matching names (Additional file 2). The totals are presented in Table 2 and show that the vast majority of vendors (80.7%) use names that match between their ethnicity and language. Among this group of matching names, 88.7% are Pwani names from the coastal inhabitants including the Dar es Salaam region, and the second largest group are Masaai names, 9.2%, a group known for their strong cultural identity. Pwani names are used by the Kwere and Zaramo, and sometimes also by the Zigua (Tables 3 and 1). Among the non-matching names, the Pwani names are predominant with 72.1%, followed by Masaai 11.1%, Zigua 7.4%, Makonde 5.9%, Pare 1.5% and Kwere 1.5%.

**Table 2:** **Cumulative totals of reported language for the 19 most common ethnospecies**

Language	#	%	Language	#	%
**Matching**	**284**	**80,7**	**Non-matching**	**68**	**19,3**
Pwani	252	88,7	Pwani	49	72,1
Zigua	5	1,8	Zigua	5	7,4
Pare	0	0	Pare	1	1,5
Masaai	26	9,2	Masaai	8	11,8
Kwere	0	0	Kwere	1	1,5
Sambaa	1	0,4	Sambaa	0	0
Makonde	0	0	Makonde	4	5,9

Left columns (Matching) show the frequencies and percentages of species that were reported in the same language as the ethnicity of the vendor. Right columns (Non-matching) show the frequency and percentages of species that were reported in a language not matching the ethnicity of the vendor and the language in which the species was reported in those cases.

**Table 3:** **Scientific names and dominant vernacular names of the studied species** *

Scientific name	Family	Dominant vernacular name (language region)	Voucher No. ^$^
*Acacia nilotica* (L.) Willd.	Fabaceae	Ol kiloriti (Masaai)	SA 24
*Acalypha ornata* Hochst. ex A.Rich.	Euphorbiaceae	Mfulwe (Pwani)	SA 39
*Afzelia quanzensis* Welw.	Fabaceae	Mkongo (Pwani)	SA 45
*Albizia anthelmintica* Brongn.	Fabaceae	Mfuleta (Pwani)	SA 19
*Bonamia mossambicensis* (Klotzsch) Hallier f.	Convolvulaceae	Mlipu (Pwani)	SA 20
*Cassia abbreviata* Oliv.	Fabaceae	Mkundekunde (Pwani)	SA 14
*Combretum fragrans* F.Hoffm.	Combretaceae	Mlama (Pwani)	SA 48
*Diospyros zombensis* (B.L.Burtt) F.White	Ebenaceae	Kasela (Pwani)	SA 41
*Holarrhena febrifuga* Klotzsch	Apocynaceae	Mmelemele (Pwani)	SA 18
*Hymenaea verrucosa* Gaertn.	Fabaceae	Mkumbi (Pwani)	SA 30
*Keetia venosa* (Oliv.) Bridson	Rubiaceae	Mkandachuma (Pwani)	SA 47
*Rapanea melanophloeos* (L.) Mez	Myrsinaceae	Mpaja (Pwani)	SA 42
*Sclerocarya birrea* (A.Rich.) Hochst.	Anacardiaceae	Mngo'ng'o (Pwani)	SA 38
*Suregada zanzibariensis* Baill.	Euphorbiaceae	Mdimu mpori (Pwani)	SA 40
*Uvaria acrantha* Miq.	Annonaceae	Msofu (Pwani)	SA 33
*Warburgia elongata* Verdc.	Canellaceae	Mwifu (Pwani)	SA 46
*Ximenia aegyptiaca* L.	Olacaceae	Mpingi (Pwani)	SA 37
*Zanha africana* (Radlk.) Exell	Sapindaceae	Mdaula (Pwani)	SA 06
*Zanthoxylum chalybeum* Engl.	Rutaceae	Mjafari (Pwani)	SA 26

*Species that were reported by 10 or more respondents. ^$^All vouchers are deposited at the herbarium of the Institute for Traditional Medicine, Muhimbili University of Health and Allied Sciences.

## Discussion

The ethnicities of the medicinal plant vendors were very diverse and reflected the ethnic diversity of Tanzania (Table 1). Many of the vendors in Dar es Salaam belong to Pwani groups, Kwere, Zaramo and Zigua (47 out of 79). In Tanga most vendors belong to the Sambaa tribe (8 of 11). In an earlier study from Tanzania, McMillen [7] discussed the difficulties in reducing ethnospecies in multiple languages (Shambaa, Maasai, Zigua and Digo), but the author focused on a limited number of species to study evolution and transmission of ethnobotanical knowledge. In both cities the principal ethnic groups in the regions were prominent among the vendors. Vendors routinely identified ethnospecies with vernacular names in their languages, but when they did not, they used the languages of the predominant groups in both cities. Many vendors knew also the name of the ethnospecies in other languages, such as their own or that of other groups. Our data show that the dominant language in which ethnospecies are reported in the Dar es Salaam and Tanga markets combined are the languages spoken in Pwani region. Nevertheless, 72.1% of vernacular names that were reported in languages that did not match the ethnicity of the vendor were reported in Pwani.

The latter language dominance could either be an artifact of interviewing in a non-native language, Kiswahili in this study, or it could show that vendors are adapting the vernacular names of the products to the mainstream cultures in these cities. Such adaptation makes sense in a market economy where instant recognition of products by passing customers is essential to securing a sale. Numerous products are packed in striking packets and other products in the market are promoted using easy to recognize names, e.g. One Plus, Extra Power or any common Swahili name not limited to a particular ethnicity such as Hakika, Bingwa, etc. Unique packaging of herbal material is a monopoly strategy in which the ownership of a product in the public domain is bound to the vendor, but without necessarily providing any added value. Packaging can however lead to concealment of the original ethnospecies.

Other aspects of folk taxonomy that are highly relevant in the discussion of this study are monotypic versus polytypic folk generics sensu Berlin [31], descriptive names and globalized plant names. Generally folk classifications have an excess of monotypic taxa, but vernacular names reported in the market could be folk generics with unreported polytypic diversity. These folk generics could constitute a number of species that are related either within the same genus or based on characteristics significant for its folk classification. Plant medicinals traded as bark, roots or powder can pose specific challenges in uncovering polytypic taxa, but DNA barcoding is transforming this and helping to uncover cryptic diversity in folk classifications [18, 19, 38]. Among the reported data we expect polytypic folk generics in important savannah and miombo woodland genera, *Acacia*, *Brachystegia*, *Isoberlinia*, *Julbernardia*, and *Zanthoxylum*, but this remains to be tested. Descriptive names are common in folk taxonomies and influence oral knowledge transmission [39]. Many descriptive names are mnemonics and confer information on the traditional use or perceived properties [39]. Leonti et al. [40] show that two ethnic groups in Mexico that have split from common ancestors over 2000 years ago share cognate plant names in their pharmacopoeias. Van Andel et al. [41] show that among Maroons in Suriname, descendants of escaped slaves, more than 40% of the vernaculars have strong resemblance in sound, structure, and meaning to African plant names for related taxa. The paper shows that Africans recognized substantial parts of the American flora, and applied their mnemonics to this new flora. In this study some of the local names are descriptive, and adoption of descriptive names from the dominant Pwani language probably helps communicating medicinals in the multiethnic reality of the medicinal plant market. In some cases local names are dropped completely in favor of globalized plant names, e.g. Echinacea, Ginseng and Ginkgo are names found in trade around the world. In Tanzania, where trade relies mainly on locally sourced plants this is only observed for *Aloe* spp., and these are often traded using the folk generic *aloevera* irrespective of the specific species or provenance.

Adaptive nomenclature and acculturation are ingrained aspects of a free market economy, but there are drawbacks with apparent risks associated to compromising one’s own ethnic indigenous knowledge for the sake of business. An example from Mexico shows that acculturation synchronizes elements from the socio-economically dominant culture by substituting similar components in the subjected society, i.e. leading to a systemic substitution of native idiom for loanwords [42]. Consequently, the peculiarities and identity of the minorities gradually diminish without proper or structured documentation.

Direct economic incentive as observed here as a driver for knowledge erosion has not been documented previously, but rapid decline of traditional culture under the influence of dominant cultures has been widely documented. Benz et al. [43] found that plant use knowledge loss among people in the Sierra de Manantlan Biosphere Reserve in Mexico was related dominant language use, i.e. people speaking the Huastec indigenous language had a more diverse and evenly shared plant knowledge than people having adopted the majority mestizo and Spanish language. Sharma et al. [44] similarly found a rapid erosion of herbal traditions among the Hajong communities in India linked to language loss. Srithi et al. [45] documented an inter-generational loss of traditional plant knowledge by Mien ethnic communities in Northern Thailand due to acculturation and interrupted knowledge transmission.

Although this study set out to seek the dominant ethnicities in the Tanzanian herbal medicine market, the interviews showed the contribution of the ethnic and cultural diversity in the current development of the traditional medicine market. Importantly, Dar es Salaam is a cosmopolitan city attracting myriads of vendors from rural areas intending to penetrate and subsist in the Pwani market. At the same time, it does not tax the vendor to relinquish their language and cultural identity by embracing the prominent market names. Tanzania is characterized by social tranquility and communities tend not to be divided by ethnic lines. However, emphasis should now be put on the necessity of proper documentation of the knowledge of the minority ethnicities that operate on the verge of contemporary market networks.

The observation of adaptation of plant names contrasts with other studies in Africa that show that vendors use vernacular names from their own language, and that their clientele often belong to their own ethnic group [20, 21, 23]. In Gabon, Towns et al. [23] found that Beninese immigrants have their own stalls, trade plants imported from Benin, using Beninese vernacular names, and sell their goods to Beninese migrant clients. In Ghana, Hausa traders were found to be selling plants with Hausa names to their Hausa clients, while Twi-speaking traders sold the same plants with Twi names to Akan people [21]. In Accra, Hausa people go to a specific market with many northern people, while Twi speaking people go to another market in the same city with Twi speaking traders [21]. In Benin, Fon, Yoruba, Dendi and Bariba traders marketed plant products in their own languages, yet in the same study it was observed that Fon and Yoruba vernacular names were mentioned even when the vendors were of different ethnicities [20]. The latter pattern is similar to that observed in this study, and reflects the need of migrant vendors to adapt their traditional names into the commercial names of the dominant ethnic group to augment trade.

## Conclusions

Knowing what species are traded commercially is the foundation for identifying threatened taxa and comparing regional and national medicinal markets, but identification of plant products in marketplaces poses challenges to monitoring commercialization of biodiversity. Rich vernacular heterogeneity due to ethnic diversity adds a level of complexion to plant identification, but this is mitigated by vernacular dominance of the predominant groups in the market towns. African cosmopolitan cities like Dar es Salaam attract people from rural areas and broadly reflect the country’s ethnic diversity. Rural entrepreneurs entering the herbal medicine trade adapt their nomenclature to optimize trade, and this malleability is observed in the variety of names of traded medicinals. However, some vendors also maintain local vernacular names, and this could reflect safeguarding cultural identity. Studying and documenting the knowledge of minority ethnicities that operate on the verge of contemporary market networks can provide valuable insights that are missed when focusing on mainstream culture and main market centers.

## Electronic supplementary material

Additional file 1: **Data S1 Master table.** (XLS 102 KB)

Additional file 2: **Data S2 Charts.** (DOCX 143 KB)
