# Supplementary material for: Vernacular dominance in folk taxonomy: a case study of ethnospecies in medicinal plant trade in Tanzania
Source: J Ethnobiol Ethnomed. 2015 Feb 19;11:10. doi: 10.1186/1746-4269-11-10 (PMC4429978; doi:10.1186/1746-4269-11-10)
Supplement: Supplementary file 2 — Additional file 2: Data S2 Charts. (DOCX 143 KB) [file 13002_2014_474_MOESM2_ESM.docx]

Ethnospecies #1 - *Zanthoxylum chalybeum* Engl.

Fig 1A. Frequency of matching vs non-matching. Fig 1B. Used language if name and language match. Fig 1C. Used language if name and language did not match.

Ethnospecies #2 – *Cassia abbreviata* Oliv.

Fig 2A. Frequency of matching vs non-matching. Fig 2B. Used language if name and language match. Fig 2C. Used language if name and language did not match.

Ethnospecies #3 - *Albizia anthelmintica* Brongn.

Fig 3A. Frequency of matching vs non-matching. Fig 3B. Used language if name and language match. Fig 3C. Used language if name and language did not match.

Ethnospecies #4 - *Zanha africana* (Radlk.) Exell

Fig 4A. Frequency of matching vs non-matching. Fig 4B. Used language if name and language match. Fig 4C. Used language if name and language did not match.

Ethnospecies #5 - *Rapanea melanophloeos* (L.) Mez

Fig 5A. Frequency of matching vs non-matching. Fig 5B. Used language if name and language match. Fig 5C. Used language if name and language did not match.

Ethnospecies #6 - *Acacia nilotica* (L.) Willd.

Fig 6A. Frequency of matching vs non-matching. Fig 6B. Used language if name and language match. Fig 6C. Used language if name and language did not match.

Ethnospecies #7 - *Bonamia mossambicensis* (Klotzsch) Hallier f.

Fig 7A. Frequency of matching vs non-matching. Fig 7B. Used language if name and language match. Fig 7C. Used language if name and language did not match.

Ethnospecies #8 - *Hymenaea verrucosa* Gaertn.

Fig 8A. Frequency of matching vs non-matching. Fig 8B. Used language if name and language match. Fig 8C. Used language if name and language did not match.

Ethnospecies #9 – *Keetia venosa* (Oliv.) Bridson

Fig 9A. Frequency of matching vs non-matching. Fig 9B. Used language if name and language match.

Ethnospecies #10 - *Warburgia elongata* Verdc.

Fig 10A. Frequency of matching vs non-matching. Fig 10B. Used language if name and language match.

Ethnospecies #11 - *Ximenia aegyptiaca* L.

Fig 11A. Frequency of matching vs non-matching. Fig 11B. Used language if name and language match. Fig 11C. Used language if name and language did not match.

Ethnospecies #12 - *Sclerocarya birrea* (A.Rich.) Hochst.

Fig 12A. Frequency of matching vs non-matching. Fig 12B. Used language if name and language match. Fig 12C. Used language if name and language did not match.

Ethnospecies #13 - *Combretum fragrans* F.Hoffm.

Fig 13A. Frequency of matching vs non-matching. Fig 13B. Used language if name and language match. Fig 13C. Used language if name and language did not match.

Ethnospecies #14 - *Suregada zanzibariensis* Baill.

Fig 14A. Frequency of matching vs non-matching. Fig 14B. Used language if name and language match. Fig 14C. Used language if name and language did not match.

Ethnospecies #15 - *Holarrhena febrifuga* Klotzsch

Fig 15A. Frequency of matching vs non-matching. Fig 15B. Used language if name and language match. Fig 15C. Used language if name and language did not match.

Ethnospecies #16 - *Uvaria acrantha* Miq.

Fig 16A. Frequency of matching vs non-matching. Fig 16B. Used language if name and language match. Fig 16C. Used language if name and language did not match.

Ethnospecies #17 - *Acalypha ornata* Hochst. ex A.Rich.

Fig 17A. Frequency of matching vs non-matching. Fig 17B. Used language if name and language match. Fig 17C. Used language if name and language did not match.

Ethnospecies #18 - *Afzelia quanzensis* Welw.

Fig 18A. Frequency of matching vs non-matching. Fig 18B. Used language if name and language match. Fig 18C. Used language if name and language did not match.

Ethnospecies #19 – *Diospyros zombensis* (B.L.Burtt) F.White

Fig 19A. Frequency of matching vs non-matching. Fig 19B. Used language if name and language match. Fig 19C. Used language if name and language did not match.
